# Dietary Safety Assessment of Flk1-Transgenic Fish

**DOI:** 10.3389/fphys.2018.00008

**Published:** 2018-01-25

**Authors:** Yalan Wei, Ling Huang, Jinghui Cao, Chenghui Wang, Jizhou Yan

**Affiliations:** Key Laboratory of Exploration and Utilization of Aquatic Genetic Resources, Ministry of Education, Department of Developmental Biology, College of Fisheries and Life Sciences, Shanghai Ocean University, Shanghai, China

**Keywords:** genetic modification, transgenic fish, dietary safety, RNA-sequencing, zebrafish

## Abstract

Genetic engineering, also called genetic modification, is facing with growing demands of aquaculture and aquatic products. Although various genetically modified (GM) aquatics have been generated, it is important to evaluate biosafety of GM organisms on the human health before entering into our food chain. For this purpose, we establish a zebrafish wild adult feeding Flk1-transgenic larvae model to examine the predatory fish's histology in multiple tissues, and the global gene expression profile in the liver. 180 days of feeding trial show that there are no significantly morphological changes in intestine, liver, kidney, and sex gonads between fish fed with Flk1 transgenic fish diet (TFD) and fish fed with regular food meal (RFM). However, a characteristic skin spot and autofluorescence increase in the theca of follicle are observed in F1 generation of TFD fish. Liver RNA-sequencing analyses demonstrate that 53 out of 56712 genes or isoforms are differentially transcribed, and mostly involved in proteolysis in extracellular region. According to GO enrichment terms these deregulated genes function in catalytic activity, steroid storing, lipid metabolic process and N-Glycan biosynthesis. These results suggest that a long term of Flk1-transgenic fish diet could alter certain metabolic pathways and possibly cause related tissue deformation. Compared to the previous reports, our feasible transgenic dietary assess system could evaluate subchronic and potential health impact of transgenic fish diet by combining multi-tissue histology and liver transcriptome analyses.

## Introduction

The demand for aquatic products is increasingly growing over the world. To increase the aquatic production, scientists have made tremendous efforts to increase growth rate, tolerance to different environment, fresh quality, and disease resistance (Maclean, 2003; Bostock et al., 2010). Since growth hormone (GH) was firstly integrated into the goldfish (Zhu et al., 1985), the genetically modified (GM) techniques have been widely applied in aquaculture experiments. More than 35 different species of fish have been tested in gene transfer studies, such as the nile tilapia *Oreochromis niloticus* (Guillén et al., 1999) and carp *Cyprinus carpio* (Yong et al., 2012). As the other GM organisms are hot and controversial topics, potential toxic effects of transgenic aquatics meanwhile become concerned. Accordingly, toxicity study is considered to be a reliable method for safety assessment.

In many experiments, low dose of transgenic carp diet does not induce biological and histological changes of Sprague–Dawley rats (Yong et al., 2012). As the GM maize grain is found to be a safe nutrient for rats (Appenzeller et al., 2009a,b), the mice fed with transgenic carp show normal endocrine production, ontogenesis and reproductive capacity in the sub generation (Zhang et al., 2000). Human volunteers also show normal clinical and biochemical parameters after ingesting the transgenic tilapia (Guillén et al., 1999). However, these studies cannot exclude the people's concern. One concern is that the food proteins and DNAs might not be completely digested so as to induce the gastrointestinal, hepatorenal and reproductive defects. The other is that, transgenic dietary may cause insertional and pleiotropic effects. The ingested foreign DNA could activate the expression of silencing genes, and eventually influent the consumer through the food chain (Dona and Arvanitoyannis, 2009). Moreover, the sex- and dose-dependent toxicity experiments should also be taken into consideration (De Vendômois et al., 2009). Fares et al., have reported that feeding the rats with GM potatoes caused the ileal surface cells degradation, swelling and multinucleation (Fares and El-Sayed, 1998).

So far there are no certain answers to the above concerns. One of the main concerns is the transgenerational and long-term influence. However, 24 multigeneration studies from 182 to 728 days did not show statistically significant differences in the parameters observed (Snell et al., 2012). Obviously taking the GM organisms as food needs more sensitive and integrative assessment of health impact. In the present study we fed wild-zebrafish (AB) line with the larvae of the same species from the stable Flk1- transgenic zebrafish line to imitate the food chain and set a model for the dietary safety assessment. The Flk1-transgenic zebrafish could express green fluorescent protein (GFP) driven by the promoter of *flk1*/*vegfr2* gene, an early endothelial marker. Initially gene *flk1* (also termed as *kdrl)* is expressed in hemangioblasts. Then Flk1 expression is stronger in developing angioblast/ endothelial precursors than in mature vessels (Liao et al., 1997). Using this model, we can track the transgenic product and evaluate its transgenerational effects on the tissue histology and organ physiobiochemical functions in the predatory fish.

## Materials and methods

### Experimental subjects

Zebrafish wild AB lines and Flk1 promoter-derived green fluorescent protein (GFP) expression construct Flk1: GFP transgenic zebrafish (Flk1-transgenic fish) were home cultured under an ambient temperature of 28.5°C as previously described (Zhang et al., 2015). For biosafety experiments, fish were processed with the tricaine methane sulfonate (MS222) or rapid cooling method for euthanasia. All experiments were performed according to the Animal Care and Use Committee guidelines of the Shanghai Ocean University (SHOU-DW-2016-004).

### Feeding process

The feeding trial was progressed according to the general guidelines of Food and Drug Administration for designing and conducting the toxicity study (Hinton, 2000). After fertilization, the AB line embryos were collected immediately and cultured in 6-well plates until growing to larvae. At the beginning of the feeding trial, 20 AB line larvae (1 month post-fertilization, 1mpf) were fed with seawater-incubated artemia nauplii (Wuli, Shandong, China) as the regular fish meal (RFM). One week later, the larvae were separated into two groups randomly. One group (F0 RFM fish) was continually fed with RFM only; the other group (F0 TFD fish) was fed with Flk1-transgenic fish larvae (48 h post fertilization; hpf) or just hatched out fries, and occasionally supplemented with small amount of RFM. Each tank (10 TFD fish) was fed with 10 transgenic larvae, twice a day. The resultant F0 TFD fish were bred to wild-type AB line to generate F1 TFD fish. F2 generation TFD fish were obtained after crossing F1 TFD with wild-type zebrafish. F1 and F2 TFD fish were fed with RFM. Similarly, the control F1 and F2 RFM fish were obtained from RFM fish.

### Histology and fluorescence observations

The tissues were removed and fixed in Bouin's fluid at 4°C for 12 h, and embedded in paraffin. 5 μm sections were cut using the microtome (LEICA RM2235, Germany) and stained with hematoxylin and eosin (HE) according to the standard protocol. As autofluorescence can provide information about morphological and pathological states of tissues and cells (Deeb et al., 2008; Wang et al., 2011), we observed HE stained sections under fluorescent microscopy after excited with different light sources (white light, fluorescent with green, blue or UV lights). The images were acquired and analyzed using ZEN 2009 software (Zeiss, Germany).

### RNA-seq data analysis

After 180 days of the feeding trial, the total RNAs were extracted from the fish liver. For each library, two liver samples were collected on ice and added into 1ml of Trizol (invitrogen). After homogenization, RNA was extracted following the manufacturer's protocol and stored at 4°C until cDNA libraries construction. For each group (TFD fish or RFM fish), two libraries were constructed for RNA-seq analysis (Genergy BIO, China). Data process and analysis were conducted as previously reported (Wang et al., 2012; Zhang et al., 2015). High-throughput sequencing generated 50–67 million raw reads for each sample using the illumina Hiseq platform (Genergy BIO, China), and more than 94% of the raw reads passed quality filtering. Briefly, the quality of the raw reads was assessed using FastQC (http://www.bioinformatics.babraham.ac.uk/projects/fastqc), and low qualities were filtered out by Trim Galore (http://www.bioinformatics.babraham.ac.uk/projects/trim_galore/). Tiltered reads were mapped to reference genome using TopHat; gene assembly and differential gene expressions were determined using Cufflinks (Trapnell et al., 2012).

The gene transcripts were expressed as Fragments Per Kilobase of transcript per Million fragments mapped (FPKM), using the Cuffnorm to calculate the expression of each sample with the corrected *P*-value of log2 (fold-change) of 1 based on the reads counts and the length of the gene mapped to the model gene (Trapnell et al., 2012). The differential expression (cut-off value) between two transcripts was defined as greater than 2-fold (log 2 ratio > 1) with *p* < 0.05. For further gene accumulation and pathway analysis we analyzed the differentially expressed genes (DEGs) by Gene Ontology (GO, http://www.geneontology.org) and the Kyoto Encyclopedia of Genes and Genomes databases (KEGG, http://www.kegg.jp/) (Ashburner et al., 2000; Kanehisa and Goto, 2000). The significant enrichment GO terms and KEGG pathways were considered with the *P*-values < 0.05 of DEGs.

## Results

### Flk1-transgenic fish diet (TFD) didn't show detrimental effects on the growth and development of the predatory zebrafish

**Figure 1:**
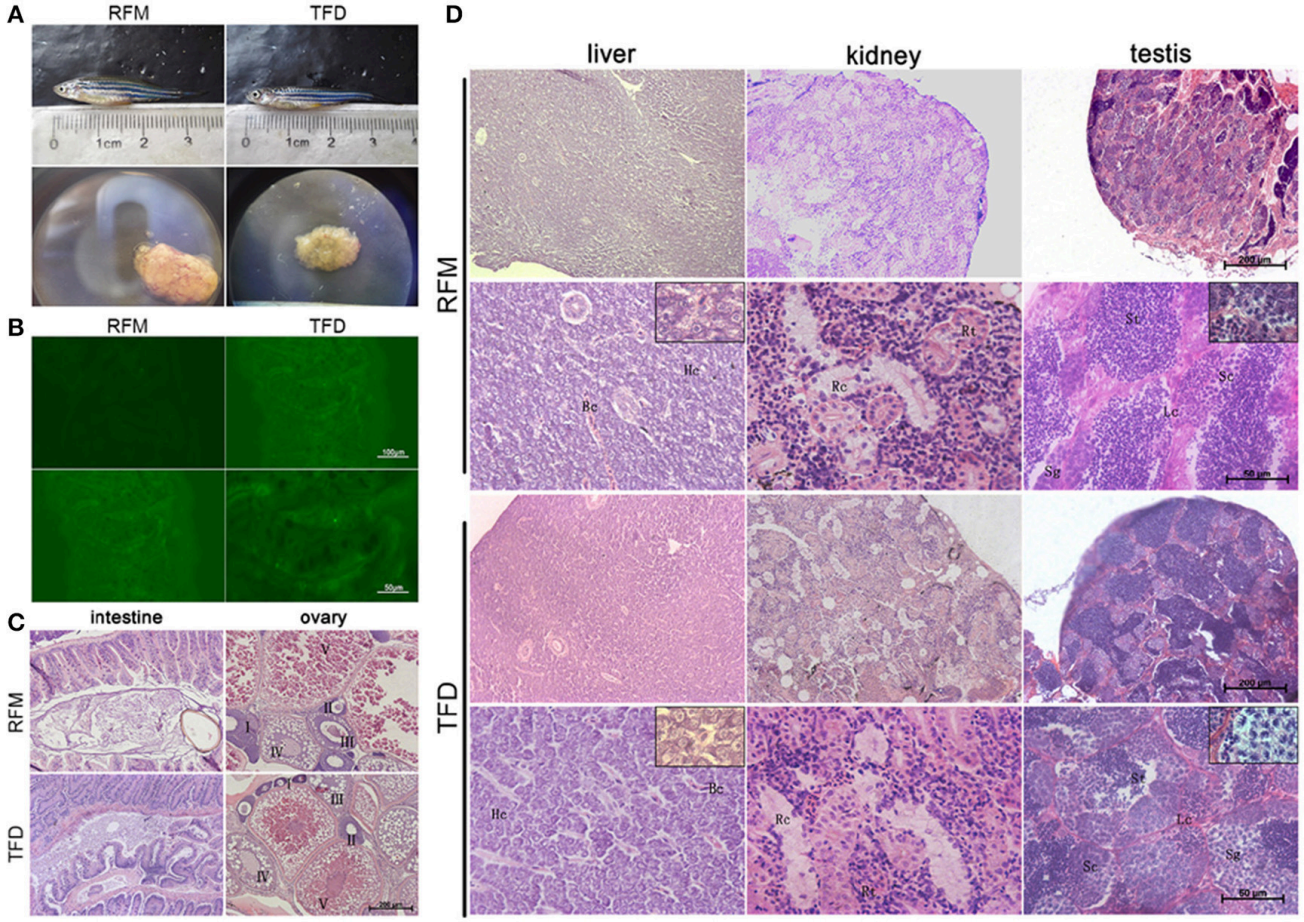
The comparisons of different characteristics between the TFD and control RFM group. **(A)** The adult fish of two groups had similar body length. The gonad size of TFD fish was smaller than to the control one. **(B)** The intestine cells of two groups didn't express GFP. Scale bars, 100 and 50 μm. **(C,D)** Histological analyses of the intestine, liver, kidney, ovary, and testis between the TFD and RFM group. I-V mean the 5 phases of oocytes, respectively. Hc, Hepatic cell; Bc, Blood capillary; Rt, Renal tubular; Rc, Renal capsule; Sg, Spermatogonia; Sc, Spermatocyte; St, Spermatid; Lc, Leydig cell; Scale bars, 200 and 50 μm.

We projected a 180 days of feeding trial to test whether eating the transgenic fish would induce any dietary harm. The feeding trial started at the juvenile period, 30–40 day post-fertilization (dpf), when the AB line zebrafish were developed big enough to eat the transgenic larvae. We randomly chose 40 fish and divided them into two groups. All fish were fed with equivalent amount of F0 TFD or F0 RFM twice per day according to the growth rate and the body weight. After 90 days, we calculated the length of the body, spawning, the growth rates and the death rates. The results showed no significant differences in the above parameters and swimming behaviors between two groups, although many F0 TFD fish had a little bit lower weight and smaller ovary than F0 RFM control (Figure 1A).

F0 TFD fish intestine showed higher intense fluorescence signal than the F0 RFM control individuals. To rule out this possibility that the digested tissues of the Flk1: GFP transgenic fish in diet may invade into the predatory intestine fish, we starved the TFD fish for 24 h to keep their intestine empty, and then fed with RFM. As a result, the fluorescence signal in the intestine became much weaker than TFD fed fish (Figure 1B). This result indicates that the Flk1:GFP protein was not intruded into the intestine but digested in the intestine cavity of the predatory fish.

The histological analyses showed that after 180 days feeding trial, the Flk1-transgenic fish diet didn't change tissue structures and cell morphology of the intestine and kidney in comparison with RFM diet (Figures 1C,D). The mucosa epithelial cells of intestine showed similar phenotypes in the thickness, crypt depth and nuclear density. The hepatic cells of two groups had the regular arrangement along with hepatic cords, and the capillaries showed a normal distribution pattern. In addition, the kidneys had regular renal tubules, capsules and mesenchymal cells. The testes had normal spermatogonium, spermatocytes, spermatids, Sertoli cells and Leydig cells. The ovaries had regular 5 phases of follicles, except small size (Figure 1C). Generally the F0 generation of TFD zebrafish has no obviously histological changes, while all individual gonads developed into testes or ovaries as described (Sun et al., 2013).

### F1 generation of TFD predatory fish developed characteristic skin and ovary defects

Having observed small ovaries in TFD fish, we then tested the fish's reproductive capacity. Seven adult female F0 TFD fish mated with wild type AB lines and produced dozens of F1 generations with normal phenotype. Neither the F1 embryos nor the F1 larvae of TFD fish expressed GFP, confirming that the Flk1-GFP transgene products were digested, and failed to be seen in the next generation of TFD fish.

**Figure 2:**
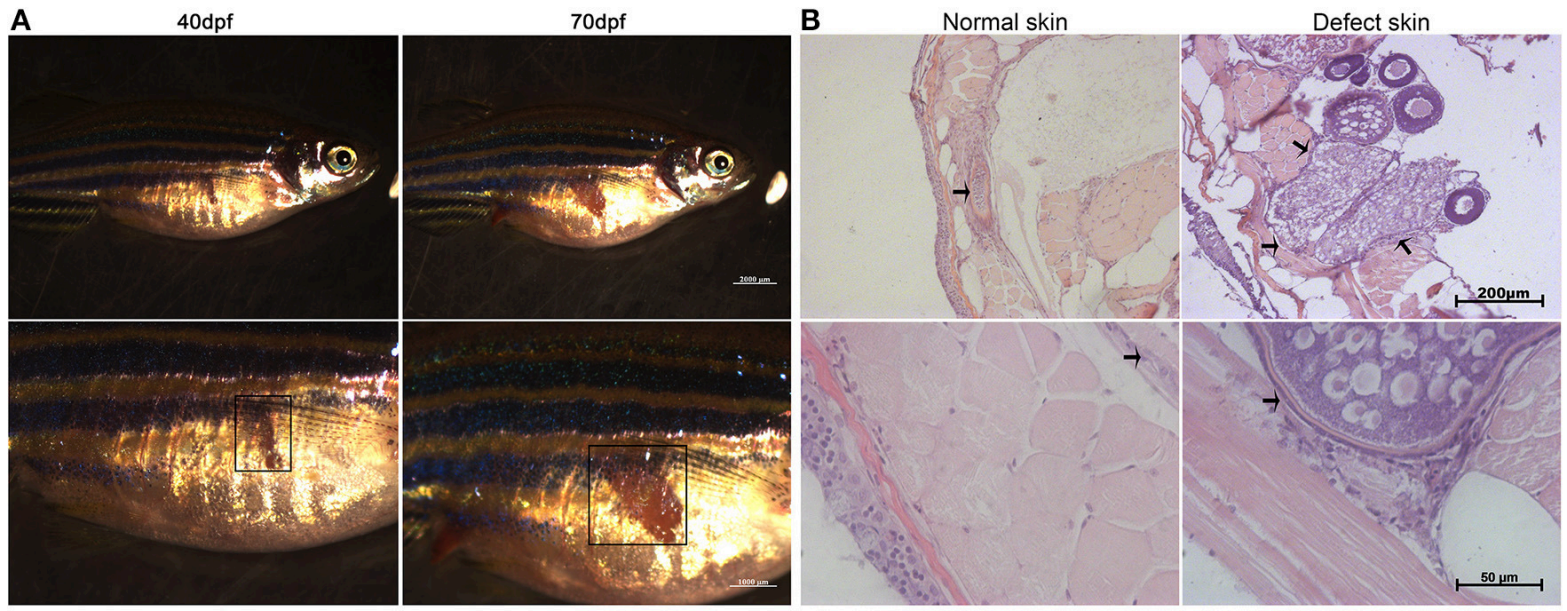
A skin developmental defect of the female F1 generation of TFD zebrafish. **(A)** The phenotypes of the F1 generation showed different sizes developmental defect. Scale bars, 2,000 and 1,000 μm. Black boxes represent the region of defect skin. **(B)** The normal and defect skin from the same F1 TFD fish. The patch and defect tissue adherent to the ovary were marked by square or arrows. Scale bars, 200 and 50 μm.

**Figure 3:**
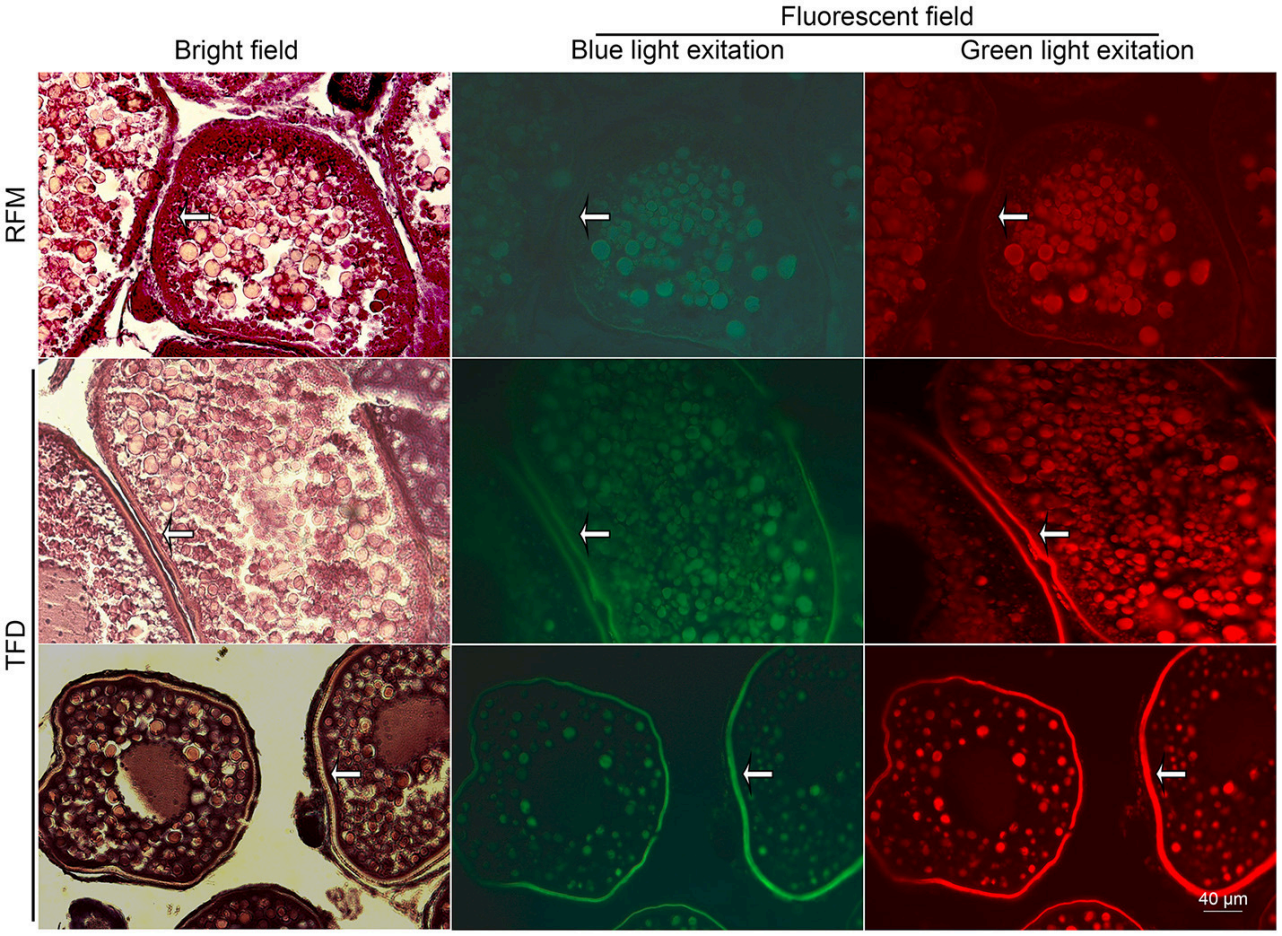
Autofluorescence of the ovarian HE stained sections in bright and fluorescent fields. White arrows represent the strengthen autofluorescence patterns in the follicle walls. Scale bars, 40 μm.

Intriguingly, most of the female F1 generation had an obvious white or deep tissue-deficient patch appeared on the left side of the abdomen skin adjacent to the ovary. Under inverted phase contrast microscope, the patch was seen in dark red color. As the fish grew, this patch became enlarged (Figure 2A). The dark red patch became pale color after the fish died. We removed the fish scale and dissected both the left and right sides of the skin to compare the tissue structure according to the histological description of zebrafish skin development (Le Guellec et al., 2003). Compared to the normal skin on the right side, the patched skin area on the left side seemed to be short of dense connective tissue in hypodermis layer, and adhered to the ovary. Consequently a part of the ovary tissue was intruded into the hypodermis (Figure 2B). The multilayer of follicle cells seen in RFM follicles became thin and partially replaced by fibrous/transparent wall in TFD follicles (Figure 3). However, the ovary size and histology of F1 generation had no significant difference compared to the control F1 RFM. An extended study did not observe the skin spot in the F2 generation of TFD fish (data not shown).

To better distinguish different cell types and stages of ovarian follicles, we combined HE staining method with fluorescent microscopy. In comparison with F1 RFM ovary, the oocytes in F1 TFD ovary showed similar fluorescence, but the follicle stroma, particularly in the theca of follicles showed stronger autofluorescence and structure clarity by the corresponding excitation light source (Figure 3). The differential autofluorescence patterns between somatic tissue and germ cells were consistent with the previous results that autofluorescence patterns could reflect the gonadal cell types and developmental stages (Wang et al., 2011). Since the TFD defected ovaries showed normal folliculogenesis, and since autofluorescence is sensitive and specific for detection of elastic and collagen fibers (Deeb et al., 2008), the intense autofluorescence along follicle walls and adjacent patched skin suggest that TFD may alter certain local connective tissue synthesis and deposition.

### The RNA-sequencing data reveal a slight difference in global gene transcription by feeding TFD

Liver is one of the most important digestive organ responsible for multiple dietary metabolisms, such as storing the polysaccharide glycogen, excreting excess nitrogen, detoxification and synthesis cholesterol fatty acids and triacylglycerols (Blann, 2014). To testify whether Flk1 fish diet interfere with the normal functions of the liver, we performed RNA-seq to analyze liver-related biological processes from F0 generation fish.

After generated all of the raw reads by high-throughput sequencing, the lower quality sequences were trimmed and removed from the reads (Supplementary Table 1). To assess the quality of sequencing data, we performed the quality control including the evaluation of the quality scores across all bases (Sanger/Illumina 1.9 encoding), GC content, the mean sequence quality (Phred Score) and the distributions of sequence length (Supplementary Figure 1). Generally the ratio of the clean reads to the raw reads in the TFD fish (94.75) was lower than that in RFM fish (99%). Similar change also occurred at the base level (Supplementary Table 1) and the quality scores across all bases (Supplementary Figure 1).

We made further statistic analysis on the nucleotide composition bias, reproducibility, the sequencing depth, strand specificity and coverage uniformity over the genome structure. Although the mapped ratios of TFD fish (89.7%) were higher than that of RFM fish (80.7%), the multiple mapped reads and splice reads were lower in TFD fish than RFM fish (Supplementary Table 2). Moreover the complete and partial novel splicing junctions were higher in TFD fish (11%) than RFM fish (9%) (Supplementary Figure 2). Collectively, a small set of gene transcripts were changed by TFD.

### A subset of metabolic genes altered after feeding transgenic fish

The differential gene expression patterns of each sample were calculated by Cuffnorm to evaluate whether the two groups had different expression levels of each gene. The transcript expression boxplot showed no significant difference between each sample (Supplementary Figure 3A). However, the principle component analysis (PCA) revealed that the expression patterns were significant different, revealed a clear difference in gene expression between TFD and RFM-fed fish. Such the difference occurred in intra-group after feeding transgenic fish for a long-term (Supplementary Figure 3B).

Genes with a fold change more than 2 and an adjusted *P*-value less than 0.05 was considered differentially expressed between TFD and RFM. Out of 56712 genes (Supplementary Table 3), 53 genes were significantly deregulated, including 31 down-regulated and 22 up-regulated genes by TFD (Supplementary Table 4). The heat map and volcano plot exhibited the cluster analysis on the genes expression difference between two groups (Supplementary Figures 3C,D).

**Figure 4:**
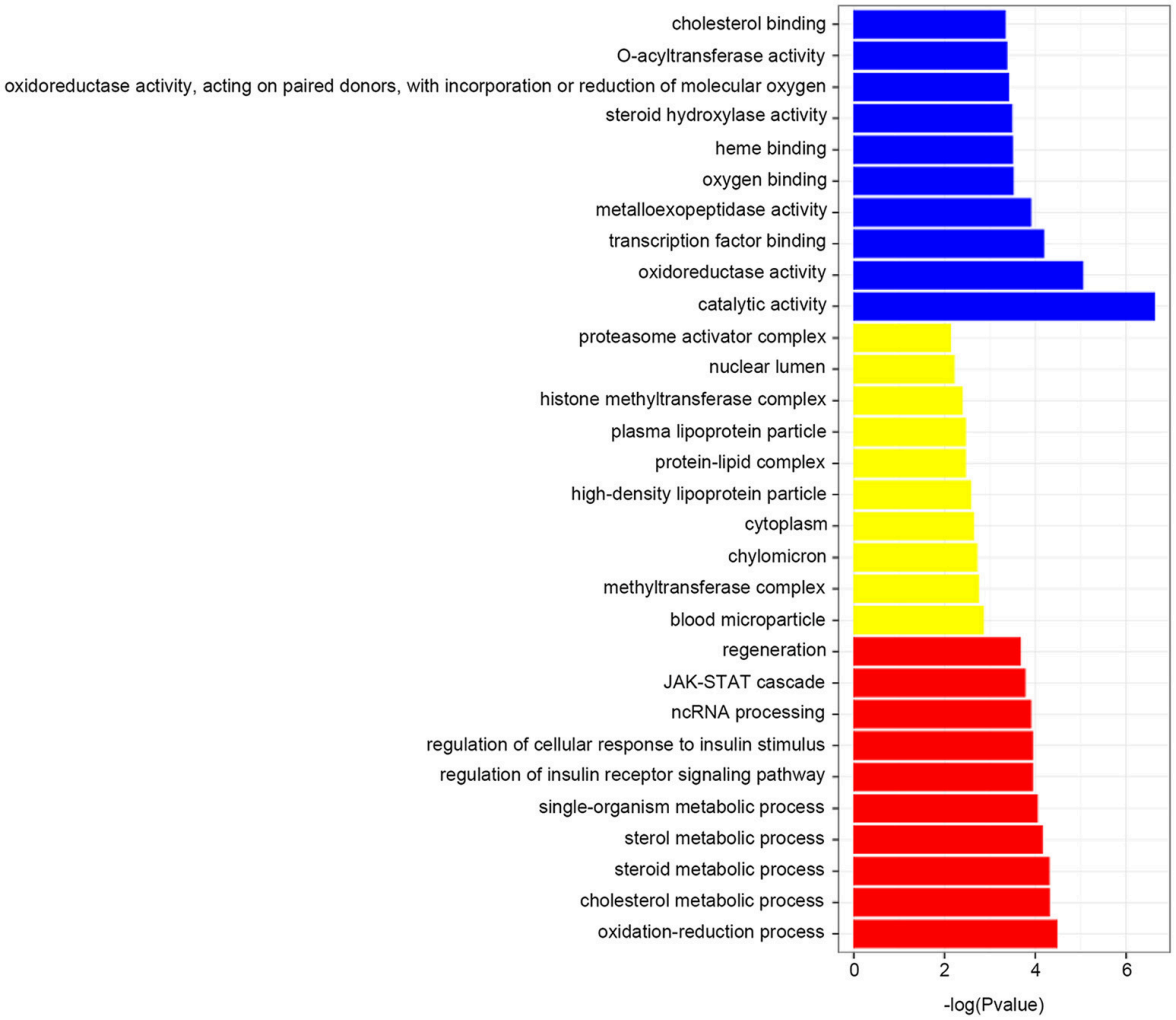
GO enrichment terms. Each biological process displays the top 10 significant terms at most, which found to be overrepresented by feeding transgenic diet.

To understand the functional classification of the differentially expressed genes, we then identified the accumulation of protein identifiers assigned to a given GO term, and compared the ratio of proteins annotated to a given GO term to the total number of proteins available from the GO database. GO categories were enriched and determined an adjusted *P*-value less than 0.05 (Supplementary Table 5). As shown in Figure 4, the enriched GO terms including catalytic activity, oxidoreductase activity, oxidation-reduction process, cholesterol metabolic process, steroid metabolic process and sterol metabolic process, were significantly found to be overrepresented by feeding transgenic diet.

The KEGG pathway enrichment analysis showed that 5 pathways were overrepresented in genes by feeding GM fish, including ECM-receptor interaction, Protein processing in endoplasmic reticulum, Lysine degradation, RNA transport, Focal adhesion and N-Glycan biosynthesis. Among them, N-Glycan biosynthesis was the most significantly enriched pathway, in which 0.25% of the genes were shown in the table list (Supplementary Table 6).

As an unbiased standard, we limited our GO term search to the significantly differential genes (Table 1). When GO enrichment analysis was performed to manifest the biological processes, two GO terms were specifically accumulated at TFD fish relative to control. One GO term was highlighted in proteolysis, and the other was unclassified. As shown in Table 1, the enriched GO terms function in cholinesterase activity, hydrolase activity, and serine-type peptidase activity. These enriched genes were mostly located in the extracellular region. Taken together, Flk1 transgenic diet changed very small parts of gene transcription, and primarily altered the extracellular matrix development of tissues and organs such as the ovary and skin hypodermis.

**Table 1:** GO enriched by feeding transgenic diet through PANTHER overrepresentation test.

**GO term**	**#**	**#**	**Expected**	**Fold enrichment**	**+/−**	*P*-value^*^
**GO BIOLOGICAL PROCESS COMPLETE**						
Proteolysis	824	9	1.22	7.4	+	8.15E−03
Unclassified	9,409	8	13.89	0.58	−	0.00E+00
**GO MOLECULAR FUNCTION COMPLETE**						
Cholinesterase activity	5	2	0.01	>100	+	4.77E−02
Hydrolase activity	2,361	13	3.48	3.73	+	3.33E−02
Serine-type endopeptidase activity	173	5	0.26	19.58	+	1.03E−02
Peptidase activity, acting on L-amino acid peptides	597	9	0.88	10.21	+	3.03E−04
Peptidase activity	619	9	0.91	9.85	+	4.10E−04
Serine-type peptidase activity	198	5	0.29	17.11	+	1.96E−02
Serine hydrolase activity	200	6	0.3	20.33	+	8.77E−04
Unclassified	8,155	5	12.04	0.42	−	0.00E+00
**GO CELLULAR COMPONENT COMPLETE**						
Extracellular space	568	8	0.84	9.54	+	1.09E−03
Extracellular region part	727	9	1.07	8.39	+	6.33E−04
Extracellular region	1,098	9	1.62	5.55	+	1.77E−02
Unclassified	9,064	9	13.38	0.67	−	0.00E+00

* *Displaying only results with P < 0.05*.

## Discussion

In the past decade, a variety of genetically modified transgenic techniques have been tried to promote the aquatic production (Forabosco et al., 2013). Growth hormone transgenic carps grow faster than the common ones and produce higher level of serum GH, which could inherit four generations stably (Zhong et al., 2009). Despite the fact that the transgenic carps showed lower swimming speed (Li et al., 2007), and the transgenic tilapia showed lower feeding motivation (Guillén et al., 1999), the transgenic aquatics require rigorous health inspection and quarantine before supplying for food markets in the future.

As one of the common model organism to study the transgenic technology (Lee et al., 2015), zebrafish have 70% genome similarities with human beings (Briggs, 2002). In this study, we established a zebrafish wild adult/Flk1-transgenic larvae model to imitate the food chain for animal eating transgenic fish, and then compared the tissue histology, and global gene expression between TFD predatory fish and RFM predatory fish through a long period and transgeneration of the feeding trial.

It was once reported that the foreign DNA fragments of the GM crops were transferred to the bacteria and remained in the human gut naturally (Halford and Shewry, 2000). To track the digestion and metabolism of the transgene products, we chose Flk1 promoter driven GFP transgenic zebrafish. In Flk1-transgenic fish, the vascular endothelial cells could express green fluorescence protein. GFP is a light-emitting protein, which chromophore is formed post-translationally from the sequence Ser65-Tyr66-Gly67 by oxidative reaction. Habibi et al. found that the ingested GFP was not degraded in gut up to 8 h and transferred to the hemolymph as a holoprotein (Habibi et al., 2002). We did observe the increased fluorescence of Flk1 transgenic fish diet in the predatory gastrointestinal tract, but the Flk1-GFP was almost thoroughly degraded within 24 h. No evidence showed that the Flk1-GFP was introduced into the intestine blood and other tissues, even though stronger autofluorescence was seen in the TFD fish ovary.

The gastrointestinal and liver are major organs for food digestions. Most of the food toxicity studies focused on the hepatic function and hepatocellular damage. Transgenic soy beans could change growth and morphology of the digestive tract of rainbow trout (Ostaszewska et al., 2005). The mice fed with genetically modified soybean might change ultrastructures and metabolism of pancreatic acinar cells, and hepocytes (Malatesta et al., 2002a,b, 2005). The rats fed with three types of GM maize also suffered from various hepatorenal toxicity (De Vendômois et al., 2009). Moreover, such GM soybean can cause the salmons a slight inflammation in the distal intestine, and even after several years it might potentially lead to cancerization (Bakke et al., 2007). It seemed that the transgenic soybean could disturb the nutrient utilization (Ostaszewska et al., 2005) and protein synthesis (Martin et al., 2003).

Different from the previous studies, we performed a long-term feeding trial through over two generations of the zebrafish, examined multiple tissues histology, and analyzed the global gene expression profile in the liver. The histological examinations showed that the liver, testis, intestine and kidney had no change in F0 TFD predators compared with F0 RFM control. However, we observed characteristic connective tissue defects in the ovaries and connected skin of many F1 generation of TFD fed zebrafish. It is not surprised to see connective tissue defects after feeding Flk1-trangenic fish, for flk1/kdrl:GFP transgene is expressed specifically in the blood vessels in hypodermis and muscle (Zhang et al., 2015).

The limited histological changes are consistent with the liver transcriptome analysis at many aspects. First, the number of affected genes is limited between the TFD subject and the control group. The annotation of each group has no significant change with approximately 90% known splicing junctions, although the principle component revealed a clear difference in gene expression. Out of 56712 genes, only 53 genes were significantly deregulated.

Second, most of the deregulated genes encode the enzymes responsible for protein (amino acids) and lipid metabolisms. Five deregulated genes (*mmp13a, mfap4, cela1, ela2*, and *p4ha3*) are involved in collagen and elastin metabolisms, and four genes (*nfkbiab, fkbp5, socs3b*, and *socs1a*) are related to pathways of cytokine or inflammatory reactions (Supplementary Table 4). Many other differential expressed genes were associated with proteolysis in extracellular matrix region. It has been found that metabolisms of proteins such as elastin and collagen, expressed by these enzyme activities, are accelerated under hypertensive vascular lesions (Yamada et al., 1986).

Third, the enriched GO terms by feeding TFD overrepresented including the catalytic activity, oxidoreductase activity, oxidation-reduction process, cholesterol metabolic process, steroid metabolic process and sterol metabolic process. The most significantly enriched pathway, N-Glycan biosynthesis, was strongly associated with the nonalcoholic fatty liver disease, hepatic fibrosis, and hepatic parenchymal cells regenerating (Lewis and Mohanty, 2010; Blann, 2014). A similar sex-dependent influence was also found in rats, which revealed that the GM corn might cause trigycerides increase in female individuals (De Vendômois et al., 2009).

Overall, the gene expression profile could explain the local skin patch and the increased autofluorescence in the follicle walls of TFD fed fish. As the extracellular matrix contributes to the autofluorescence emission more than the cellular components (Deeb et al., 2008), our data suggest that Flk1-GFP TFD could induce changes in certain metabolic pathways, leading to the subchronic soft tissue deformations.

## Conclusion

Flk1:GFP transgenic zebrafish expresses green fluorescent protein (GFP) under the flk1/vegfr2 promoter (an early endothelial marker), and exhibits a green fluorescent vasculature. After feeding Flk1:GFP transgenic larvae, the predatory fish show developed soft tissue defects in the ovary and adjacent skin hypoderm in the F1 generation. However, this defect was uninheritable. Compared to the previous reports reviewed by Snell et al. (2012), our feasible transgenic dietary assess system evaluates subchronic effects of transgenic fish at both multi-tissue histology and transcriptome level, and identifies the genotype-phenotype correlations.

## Author contributions

JY conceived the idea. JY, YW analyzed the data and wrote the manuscript. YW, LH, and JC performed the experiments, and analyzed the data. CW supervised the project and contributed to the final version of the manuscript.

### Conflict of interest statement

The authors declare that the research was conducted in the absence of any commercial or financial relationships that could be construed as a potential conflict of interest.

## Supplementary material

Supplementary Figure 1 RNA-sequencing quality control.

Supplementary Figure 2 Nucleotide composition bias and reproducibility.

Supplementary Figure 3 Different gene expression levels and functional analysis between TFD and control groups. **(A)** The transcript expression boxplot of each sample. **(B)** The different PCA of gene expression between the TFD and control samples. **(C)** Heat map of liver tissues from TFD and control subjects (Distance metric: Pearson correlation; Linkage rule: Average Linkage). **(D)** Volcano plot of different gene expression between two groups.

Supplementary Table 1 Statistics for the raw reads quality clean.

Supplementary Table 2 High-quality reads mapped against reference genome.

Supplementary Table 3 Differential gene expression between RFM and TFD fish. Value_1 and value_2 represent the mean FPKM expression of RFM and TFD samples. Log2 (fold_change) is the natural logarithm of FPKM of sample 2 to sample 1. The negative value means that sample 1 up-regulated expressed to sample 2 relatively, vice versa. Test_stat is the significance of the observed change in FPKM. The false discovery rate of *p-*value and *q-*value were corrected by Benjamini-Hochberg correction.

Supplementary Table 4 Significantly deregulated genes between RFM and TFD fish.

Supplementary Table 5 Significant enrichment of GO terms by Benjamini & Hochberg multiple tests.

Supplementary Table 6 Induced KEGG signaling pathways.

## Abbreviations

DEGs: differentially expressed genes
dpf: day post-fertilization
FPKM: Fragments Per Kilobase of transcript per Million fragments mapped
GFP: green fluorescent protein
GH: growth hormone
GM: genetically modified
GO: Gene Ontology
HE: hematoxylin and eosin
hpf: hours post-fertilization
KEGG: Kyoto Encyclopedia of Genes and Genomes
mpf: month post-fertilization
MS222: tricaine methanesulfonate
PCA: principle component analysis
RFM: regular fish meal
TFD: transgenic fish diet.
