# Supplementary figures and images for: Dietary Safety Assessment of Flk1-Transgenic Fish
Source: Front Physiol. 2018 Jan 25;9:8. doi: 10.3389/fphys.2018.00008 (PMC5788912; doi:10.3389/fphys.2018.00008)

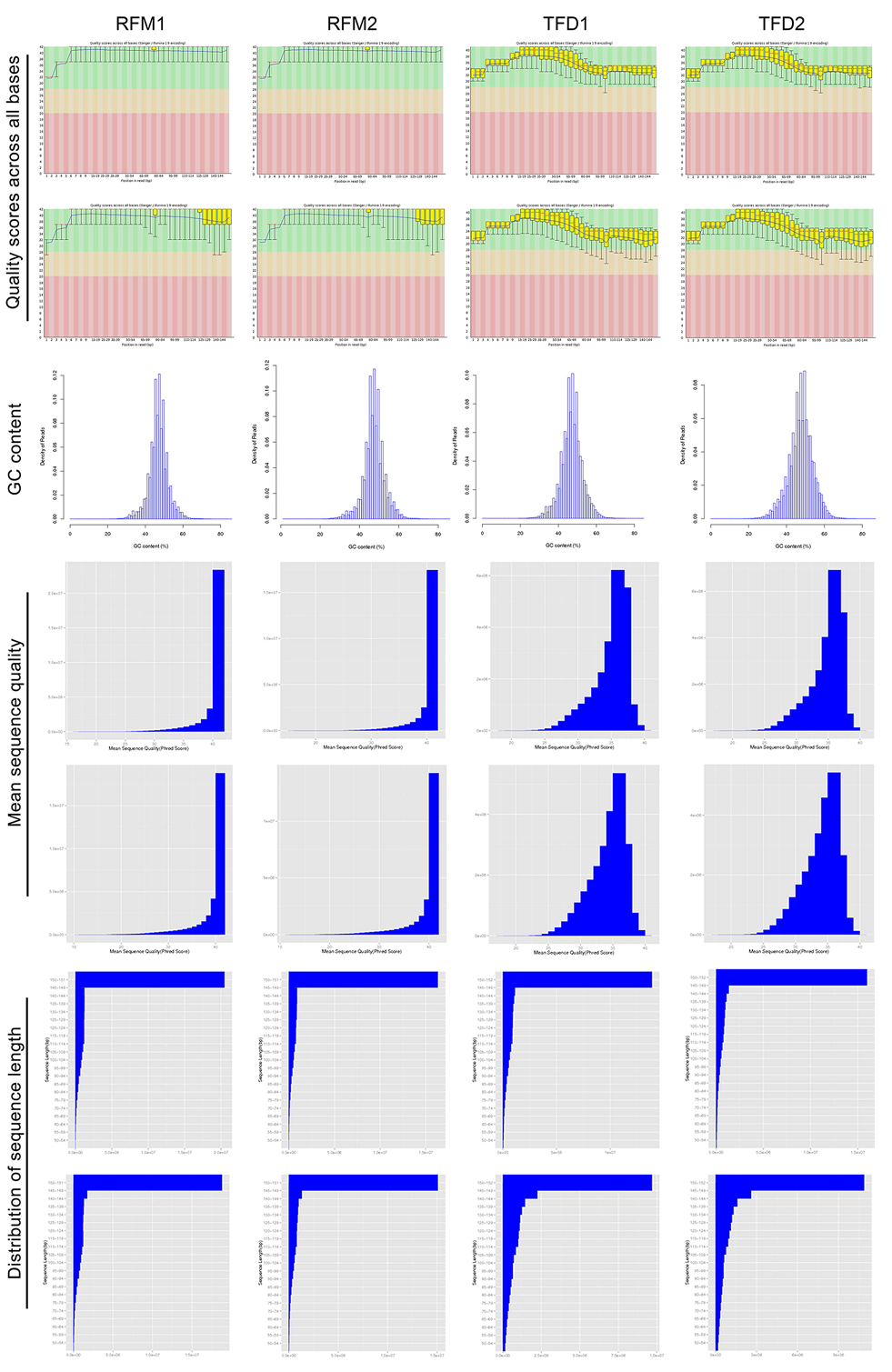

Supplement: Supplementary Figure 1 — RNA-sequencing quality control. [file Image1.TIF]

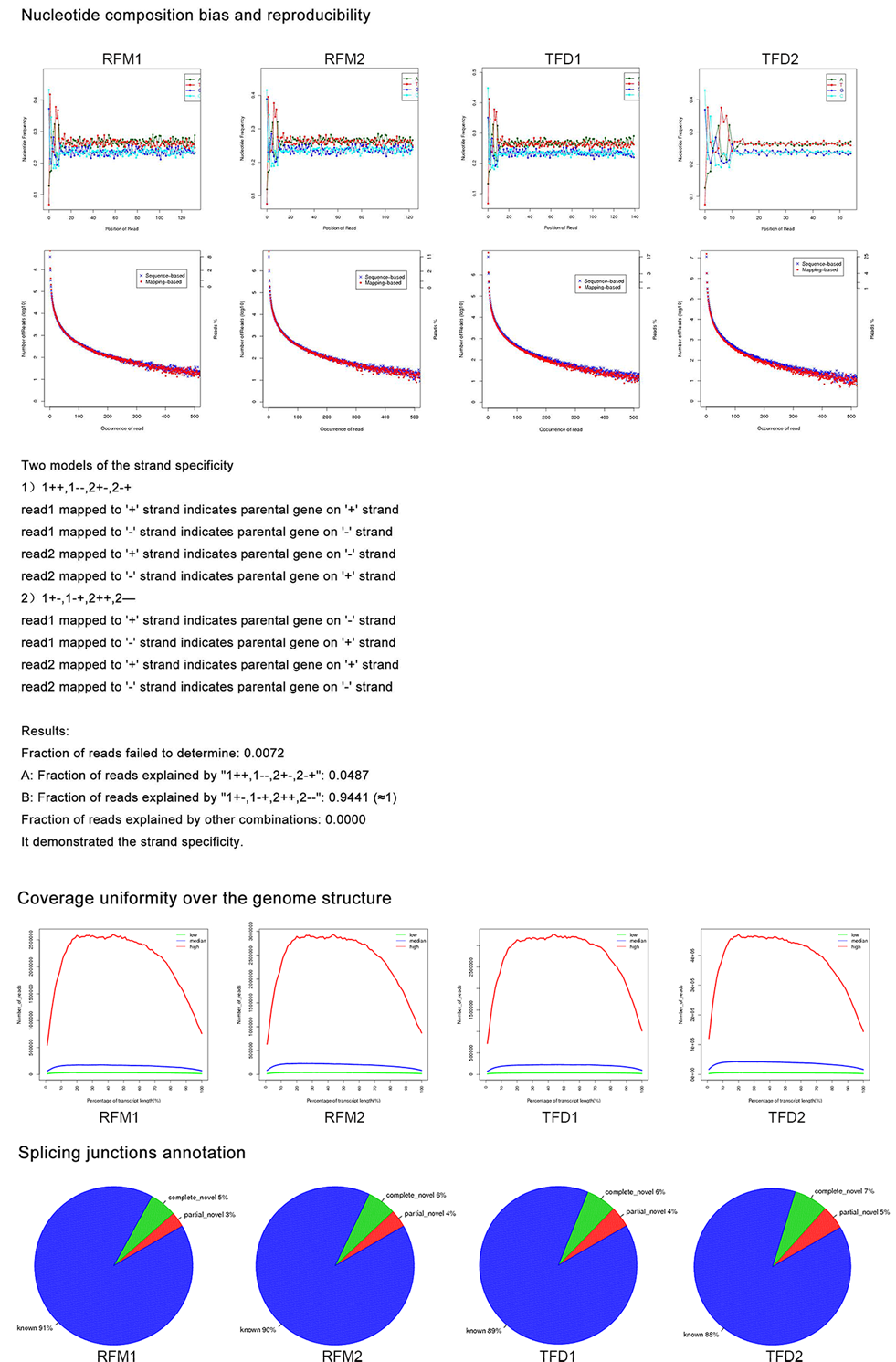

Supplement: Supplementary Figure 2 — Nucleotide composition bias and reproducibility. [file Image2.TIF]

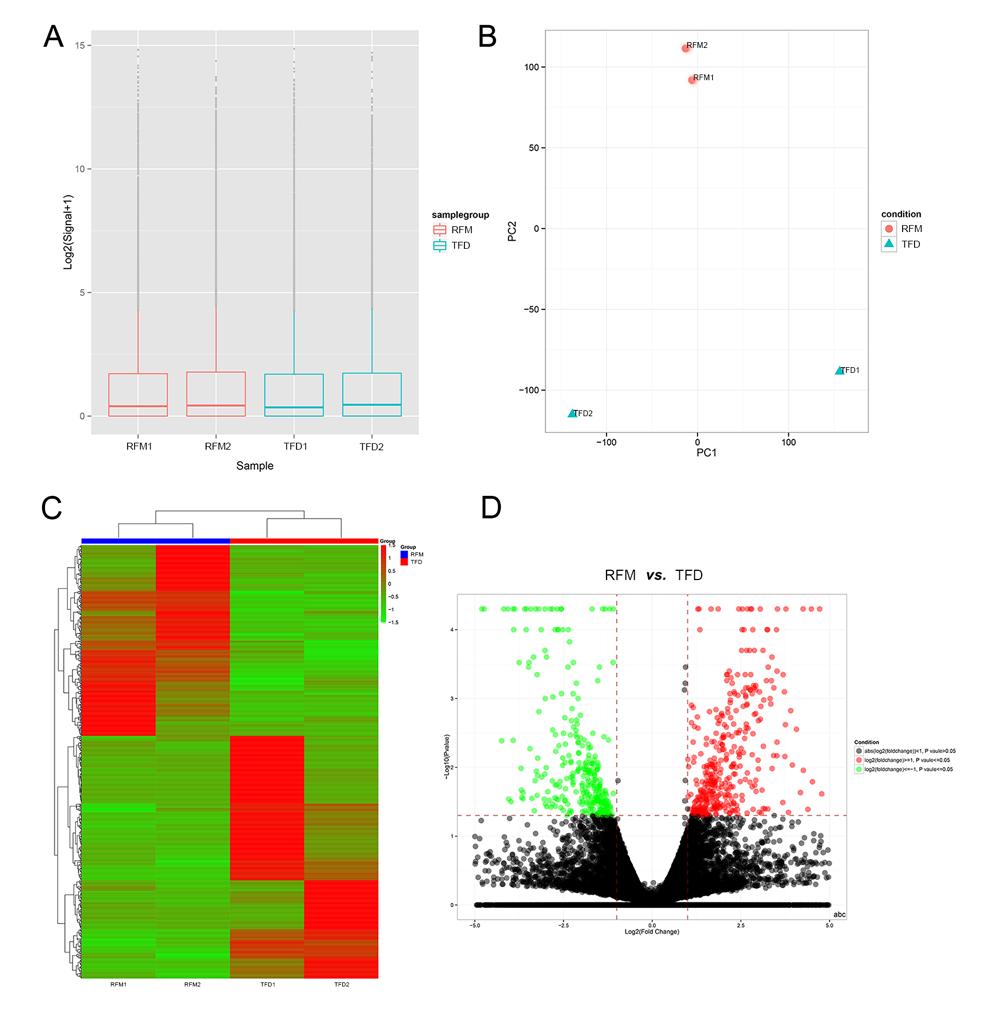

Supplement: Supplementary Figure 3 — Different gene expression levels and functional analysis between TFD and control groups. (A) The transcript expression boxplot of each sample. (B) The different PCA of gene expression between the TFD and control samples. (C) Heat map of liver tissues from TFD and control subjects (Distance metric: Pearson correlation; Linkage rule: Average Linkage). (D) Volcano plot of different gene expression between two groups. [file Image3.TIF]
